# Healthcare professionals’ information need related to antiseizure medication use in breastfeeding patients with epilepsy. Retrospective analysis of enquiries to Norwegian medicines information and pharmacovigilance centers

**DOI:** 10.1016/j.ebr.2023.100629

**Published:** 2023-10-25

**Authors:** Sunniva Reitan Riibe, Kristine Heitmann, Jan Schjøtt, Bettina Riedel

**Affiliations:** a Department of Clinical Science, University of Bergen, 5021 Bergen, Norway; b Department of Medical Biochemistry and Pharmacology, Haukeland University Hospital, 5021 Bergen, Norway; c Regional Medicines Information and Pharmacovigilance Centre (RELIS Vest), Haukeland University Hospital, 5021 Bergen, Norway; 1 Sandnessjøen Helgeland Hospital, Prestmarkveien 1, 8800 Sandnessjøen, Norway

**Keywords:** Epilepsy, Antiseizure medication, Antiepileptic drug, Breastfeeding, Risk perception, Healthcare professional

## Abstract

**Background:**

Safety information of antiseizure medication (ASM) during breastfeeding is scarce and conflicting. We aimed to identify characteristic traits of safety concerns among healthcare professionals by reviewing enquiries to the Norwegian Regional Medicines Information and Pharmacovigilance Centres (RELIS).

**Method:**

Enquiries related to breastfeeding, epilepsy, and ASM identified by their ATC-numbers were retrieved from the RELIS database of question-and-answer pairs (QAPs) by combining indexed and Boolean database searches and manual inspection. 112 QAPs were analyzed retrospectively using descriptive statistics.

**Results:**

Hospital-employed physicians and nurses were puzzled by ambiguous or conflicting drug information advice and called for general information about the compatibility of an ASM with breastfeeding, mainly related to lamotrigine and levetiracetam. Other enquiries were related to co-medication with other drugs, mainly antidepressants. Half of the enquiries were posed after birth, 12 of these motivated by suspected adverse events in the infants.

**Conclusion:**

Healthcare professionals with acknowledged high competence in the topic were uncertain about the prevailing safety information of ASM during breastfeeding. The fear to harm the infant may lead to the decision not to recommend breastfeeding. Future information strategies should aim to reach these professions, and support their information need on this topic.

## Highlights

• Hospital-employed physicians and nurses were the main enquirers.
• Most enquiries were related to lamotrigine and levetiracetam.
• Many enquiries were due to ambiguous or conflicting drug information.
• Some enquiries concerned co-medication and adverse events.
• Half of all enquiries were made after the infant was born.

## 1 Introduction

Breastfeeding has well-established short and long-term health benefits for both child and mother. For breastfed infants, these benefits comprise a large variety of health aspects, including reduced infant mortality, fewer infectious diseases, and a risk reduction for atopic dermatitis and asthma [1]. They were less likely to become obese, develop diabetes and childhood leukemia [1], [2]. Recent evidence also supports the notion that improved cognitive outcome is related to breastfeeding [3]. In mothers, the advantages of breastfeeding comprise a reduced risk for breast and ovarian cancer in addition to type 2 diabetes [4]. Breastfeeding may also strengthen the emotional bond between the mother and child [5], [6].

Breastfeeding rates vary between countries and ethnic groups but are lower in persons with epilepsy (PWE) compared to healthy individuals and in PWE using antiseizure medication (ASM) compared to PWE that do not [7], [8], [9], [10]. Data from Norway showed that 72 % and 46 % of PWE breastfed their infant one and six months after delivery compared to 83 % and 56 % of the controls, respectively [7].

Several studies support the notion that PWE using ASM should be encouraged to breastfeed, provided careful monitoring of the infant [11], [12], [13]. The overall drug exposure was low in breastfed infants of PWE receiving ASM [14], and harmful effects of breastfeeding during maternal ASM therapy in children who were also previously exposed to ASMs during their mothers’ pregnancy were not detected [7], [12], [13]. Whereas breastfeeding per se is strongly recommended, data related to ASM-exposed infants’ safety is scarce and often based on small groups of patients, depending on the ASM used by the lactating mother [15].

The decision to breastfeed is related to a variety of aspects [9], [10]. These may include healthcare professionals’ supportive or discouraging attitude towards breastfeeding contributing to a higher breastfeeding rate or to beastfeeding termination [9]. Discouragement may be based on a biased risk perception due to inconsistent or ambiguous recommendations in different sources of information [16], limited access to appropriate information sources for decision support, or limited safety information related to the ASM in question [17], [18].

In Norway, a network of four Regional Medicines Information and Pharmacovigilance Centres, collectively named RELIS, is contributing to rational use of drugs in Norway [19]. The centres are associated with clinical pharmacology units in regional university hospitals, and pharmacists and physicians in clinical pharmacology with expertise in searching and critical evaluation of literature constitute the staff in the centres. Through the question-and-answer service healthcare professionals in Norway can get manufacturer-independent advice regarding the use of drugs adapted to the medical problem of the individual patient. The employees on duty answer all types of enquiries. Complex enquiries are discussed with several colleagues and the written answers are quality checked and co-signed by a second employee. Approximately one third of all enquiries to RELIS are related to medicines use in pregnancy and lactation [20]. In this aspect, ASM was among the drug categories the most frequently asked about [21]. Thus, the general competence related to drug use in breastfeeding among pharmacists and physicians at RELIS is high. After introduction of SafeMotherMedicine in 2011, a public service in Norway which gives individual advice about drugs to pregnant and breastfeeding persons, the employees have obtained a more extensive experience in answering questions related to the topic [22].

The aim of this study was to unveil encountered challenges concerning breastfeeding when PWE use ASM described through enquiries from healthcare professionals, received by RELIS. The findings may help to identify obstacles to breastfeeding in PWE and can be valuable in the development of an adapted information strategy for healthcare professionals.

## 2 Material and methods

### 2.1 Material

The RELIS database contains more than 55 000 spontaneously asked questions by healthcare professionals regarding drugs. The enquiries are often short clinical narratives and are answered by pharmacists and physicians with competence in pharmacology, literature search, and appraisal of the literature [20], [21]. Thus, enquiries are stored as question–answer pairs (QAPs) in the RELIS database and the majority is freely available on RELIS’ website [19].

All the QAPs in the RELIS database are indexed with searchable categories (e.g., BREASTFEEDING) [21]. Healthcare profession of the enquirer (physician, pharmacist, dentist, nurse or other), and place of employment (in or outside hospital) are indexed for each QAP. The medications in each QAP are registered according to their generic name, trade name and ATC-number following the WHO’s Anatomical Therapeutic Chemical (ATC) classification system [23]. Antiseizure medications (ASMs), except clobazam, are classified in ATC-group N03, whereas the latter has ATC-number N05B A09. It is possible to refine the search using Boolean operators (e.g., AND, OR), and to specify time periods for the search.

### 2.2 Analysis of question and answer pairs

We examined QAPs from the RELIS database in the period from November 1995 to April 2021. We used the search strings “N03 AND breastfeeding”, and “N05B A09 AND breastfeeding”. Each retrieved QAP was manually examined by one author (SRR). QAPs concerning epilepsy, in addition to ASM and breastfeeding, were included. If the treatment indication was not specifically mentioned, the QAPs were examined by three authors (SRR, KH, BR) in order to decide whether a QAP should be included. This assessment was based on the daily dose stated in the question compared to the recommended dose for treatment of epilepsy, type of co-medication, if any, as well as the clinical narrative.

The data retrieved by the search strategy was subject to a preliminary review which, together with a previous study [21], made the basis for the protocol, please see supplemental file Table A.1 and Table A.2.

For included QAPs, a descriptive analysis was carried out, based on the collection of the following data: Characteristics of the healthcare professional submitting the question (physician, nurse, pharmacist, other; workplace if physician: hospital, outside of hospital), the specific ASM mentioned and the use of other medication in addition to ASM (according to the ATC-system), topic of the question (categorized according to Table 1) as well as timing of the question relative to birth and breastfeeding initiation. Finally, we investigated whether there was any difference between physicians and the group of other healthcare professionals with respect to the reason for the question, and the timing in relation to birth and initiation of breastfeeding.

The answers from RELIS were analyzed with respect to the conclusion, that is, whether the given ASM was compatible with breastfeeding. The conclusions were divided into categories “compatible with breastfeeding”, “take precautions”, “not recommended” and “no conclusion”. If several ASMs were reviewed and/or if ASM were reviewed together with other medication, the conclusion for each medication was registered as well as the overall conclusion.

### 2.3 Statistical analysis

Descriptive analyses were performed to uncover frequencies of the different categories of enquiries according to type of enquirer and enquiry, and the time point of the enquiry in relation to birth and breastfeeding initiation. For comparison of data between groups of enquirers, data from healthcare professionals other than physicians were grouped. The data were analyzed using IBM SPSS Statistics Version 27.0.1.0 (IBM Corp, Armonk, NY, USA).

### 2.4 Ethics

QAPs are anonymously stored in the RELIS database, patient data of sensitive character are not included in this study. Due to the anonymous data material, need for ethics approval by The Regional Committees for Medical and Health Research Ethics Western Norway, REK West, and consent to participate, was deemed unnecessary. This is in accordance with the research guidelines at Haukeland University Hospital, Helse Bergen Health Authority, the Health Research Act and the Personal Data Act [24].

## 3 Results

### 3.1 Description of the material

**Fig. 1:**
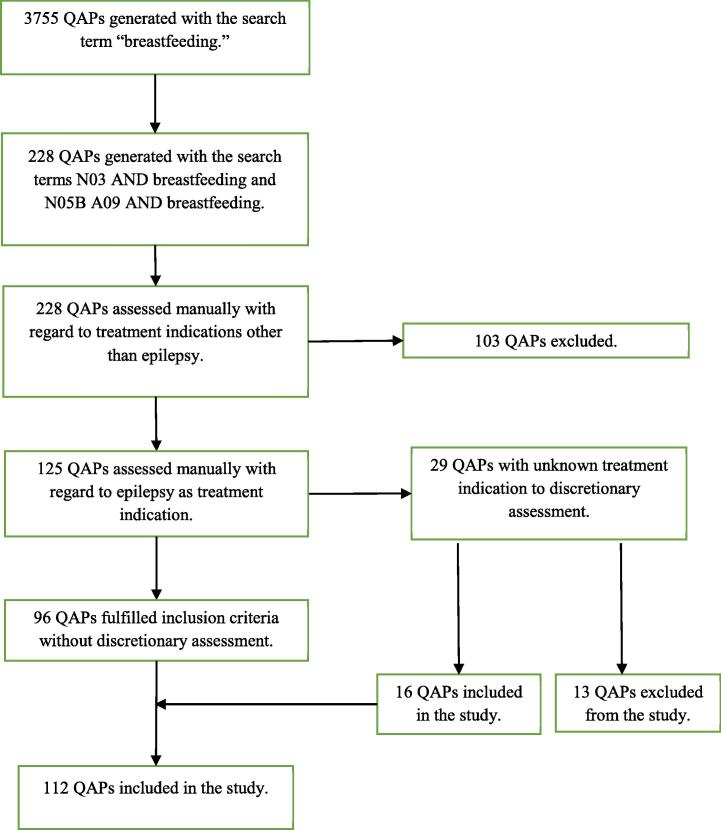
Flowchart of included and excluded QAPs. QAPs = question and answer pairs.

The RELIS database contained 55 304 QAPs by the end of December 2021 of which 228 were retrieved to be manually investigated to fit all the inclusion criteria as stated in the study protocol. Finally, 112 questions related to ASM, breastfeeding and epilepsy were eligible for inclusion into the study, as shown in Fig. 1. A total of 116 QAPs were not included into the study because the enquirer was a patient (0.9 %, n = 2), the treatment indication was other than epilepsy, such as mental illness (34.2 %, n = 78), pain (6.1 %, n = 14) or other disease (5.2 %, n = 12), or we could rule out with certainty that the enquiry concerned epilepsy (4.3 %, n = 10).

### 3.2 Enquiries and enquirers

Table 1 shows the main categories of the enquiries received by RELIS. The most frequently asked questions were based on an overall safety concern and called for general information about the compatibility of the ASM in question with breastfeeding. Some enquirers specifically highlighted ambiguous drug information of a drug’s compatibility with breastfeeding, or even the recommendation not to breastfeed to be the reason for their information need. They had obtained such advice from prescription texts in The Summary of Product Characteristics (SPC) which is a legal product approved of the marketing authorisation of each medicine by the Norwegian and European medicines agencies, or in The Norwegian Pharmaceutical Product Compendium (PPC) which prescription texts are based on the SPC but presented in a more user-friendly structured format. Other questions were motivated by the enquirers’ concern that the use of two or more drugs, including co-medication with several ASMs would not be safe for the breastfed infant, or that symptoms observed in the breastfeed infant might be related to the mothers’ use of one or several drugs.

**Table 1:** Topics of enquiries.

**Topics**	**Example**^1^	**N**	**%**^2^
**General enquiry**			
Requests general information regarding specific ASMs	Is the use of lamotrigine compatible with breastfeeding?	59	52.7
Requests updated assessment from RELIS	Is there more updated information, other than a RELIS article from 2007, on malformation risk and breastfeeding with ethosuximide treatment?	6	5.4
Not recommended or ambiguous product information advise^3^	Is there any literature on whether perampanel is compatible with breastfeeding, as manufacturer writes that there is a lack of experience, and that risk cannot be ruled out?	12	10.7
Other	Can lamotrigine affect milk production?	4	3.6
**Co-medication**			
Uncertain due to treatment with several ASMs	Is the combined use of levetiracetam and carbamazepine compatible with breastfeeding?	9	8.0
Uncertain due to treatment with ASM in combination with other medication	Is it safe to breastfeed while using levetiracetam and escitalopram?	9	8.0
**Suspected adverse drug reaction in breastfed infant**	Could the mother’s use of lamotrigine cause the child’s twitching?	13	11.6

ASMs = antiseizure medications, RELIS = Regional Medicines Information and Pharmacovigilance Centres.

1 Details about mother, child and dose omitted.

2 Percent of 112 enquiries.

3 The Norwegian Pharmaceutical Product Compendium or The Summary of Product Characteristics.

Physicians represented 53.6 % (n = 60) of all healthcare professionals who requested information from RELIS on the compatibility of breastfeeding with ASM-use in PWE. Most were employed in a hospital (83.3 %, n = 50), whereas the other physicians worked outside the hospital (16.7 %, n = 10), either as general practitioners, gynecologists or as physicians at Maternity and Child Health Care Centres. A substantial part of the enquiries was made by nurses (35.7 %, n = 40), while enquiries from pharmacists and other healthcare workers accounted for 4.5 % (n = 5) and 6.3 % (n = 7) of all enquiries, respectively. Within the specific categories of enquiry, physicians and other healthcare professionals were equally represented among the enquirers, but physicians more often specified that their need for decision support was motivated by ambiguous or restricted product information advice in the PPC or the SPC (Table 2).

**Table 2:** Association between different occupational groups of healthcare professionals and enquiry topics^1^

**Topic**	**Occupation**			
	Physcian		Other healthcare professionals^2^	
	N	%	N	%
General enquiry	35	31.3	34	30.4
Not recommended or ambiguous product information advice^3^	11	9.8	1	0.9
Co-medication	8	7.1	10	8.9
Suspected adverse drug reaction in breastfed infant	6	5.4	7	6.3

1 Percent of 112 enquiries.

2 Nurses, pharmacists, other healthcare professionals.

3 The Norwegian Pharmaceutical Product Compendium or The Summary of Product Characteristics.

### 3.3 Time point of enquiry

**Fig. 2:**
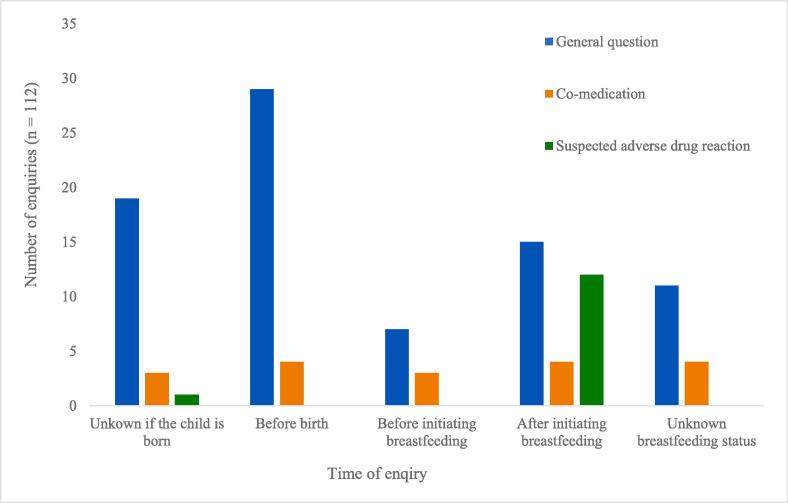
Category of enquiry submitted to RELIS at the different time points in relation to birth and initiation of breastfeeding. RELIS = Regional Medicines Information and Pharmacovigilance Centres.

The distribution over time of the different questions in relation to birth and breastfeeding are given in Fig. 2. Half of all included enquiries (50 %, n = 56) were asked after the child was born, and 31 of these (55 %) were asked even after the patients had started to breastfeed their infant. Among the enquiries made after birth, ten (5 %) were made before the mothers had started to breastfeed their infant. Thirty-three questions (29.5 %) were asked before the child was born, and two of them were posed even before conception. The remaining enquiries (20.5 %, n = 23) contained no information about the timing in relation to birth, the child’s age, state of health, or whether breastfeeding was initiated.

General enquiries related to the compatibility of breastfeeding in infants of PWE using ASM were prevailing among all enquiries at all time points, that is before and after birth, and were also unrelated to the initiation of breastfeeding. This was also true for enquiries related to the breastfed infants’ safety during the mothers’ use of several drugs, including several ASMs. Of all enquiries related to the breastfed infants’ reduced health status suspected to be associated with the mothers’ ASM use (n = 13), 8 of them were posed before the child was 2 months old or younger (Table 3).

### 3.4 Drugs

**Fig. 3:**
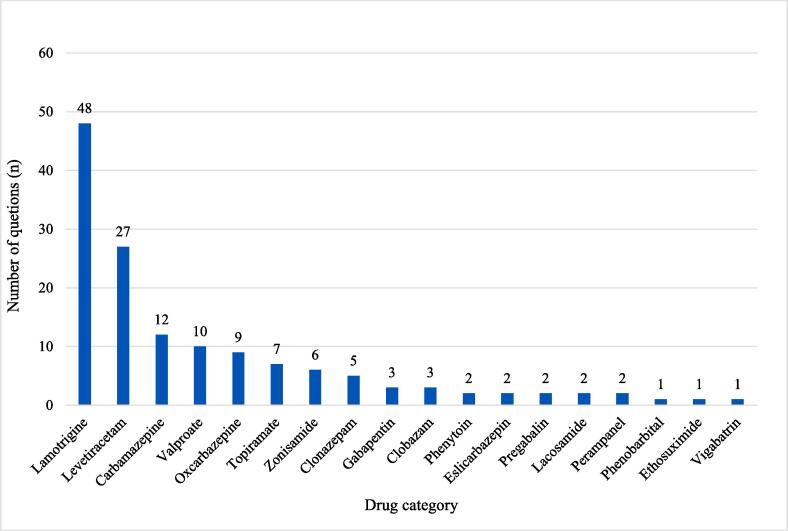
Data labels indicate the number of times the specific drug was subject for an enquiry. Some enquiries involved more than one drug.

The frequencies of ASMs mentioned in the enquiries are shown in Fig. 3. Enquiries related to monotherapy with lamotrigine were most prevalent (33.0 %, n = 37), followed by those related to monotherapy with levetiracetam (13.4 %, n = 15) and those regarding lamotrigine and/or levetiracetam in polytherapy with one or more ASMs (17.8 %, n = 20). Lamotrigine and levetiracetam were also the most often mentioned ASMs when the enquirers wondered whether the infant’s symptoms could be drug related. Enquiries concerning possibl adverse drug rections in the infants are given in Table 3. Enquiries related to treatment with other ASMs than levetiracetam and lamotrigine, accounted for about one third of all enquiries (35.7 %, n = 40). In 17 enquiries (15.2 %), other drugs were mentioned in addition to ASMs. Drugs belonging to ATC group N were the most prevalent, with antidepressants (ATC group N06A) accounting for the largest proportion of enquiries, (7.1 %, n = 8).

### 3.5 Drug safety and answers from RELIS

In response to 95 of 112 enquiries (84.8 %), RELIS advised that PWE could breastfeed if certain precautions were taken, such as observation of the infant with specific symptoms (e.g., sedation and weak suction) and analysis of drug concentration in the infant’s serum. In seven (6.3 %) of the QAPs, RELIS advised against breastfeeding, due to co-medication (1.8 %, n = 2), the infant’s impaired state of health (0.9 %, n = 1), insufficient documentation of effects on breastfed infants as well as transfer of the drug into breast milk (3.6 %, n = 4). In response to four (3.6 %) enquiries, all of which dealt with valproate, the patients were recommended to breastfeed, and no precautions were given. In six (5.4 %) cases, RELIS did not give any specific advice on breastfeeding. Among the 56 enquiries made after the infant was born, 12 (21.4 %) were made based on adverse events in the breastfed infants that were suspected to be related to the drugs taken by the lactating patient. Information on the child’s state of health was sparse otherwise or lacking in 33 (58.9 %) postnatally asked questions. Table 3 shows the suspected drugs involved, the infants’ age, the observed symptoms, and the specific advice from RELIS. Several drugs were mentioned in three enquiries.

**Table 3:** Enquiries related to suspected adverse drug reactions in breastfed infants, the patients’ medication and advice from RELIS (n = 13)1.

**Adverse effects in breastfed infant**	**Drug(s)**	**Advice from RELIS**^2^
Two months old infant. Enquiry about the impact on the child’s stool	Levetiracetam, phenytoin, gabapentin	Be observant for signs of sedation or poor weight gain. If necessary, measure the child’s serum concentration of the drug. Breastfeed at a time when the drug concentration in breast milk is the lowest.
Four weeks old infant. Sedated.	Carbamazepine	Measure the child’s serum concentration of the drug. Breastfeed at a time when the drug concentration in breast milk is the lowest. If necessary, consider interrupting breastfeeding.
One week old infant. Drowsy.	Phenobarbital	Assess the child’s symptoms on a regular basis.
Three months old infant. Up to ten watery stools daily.	Topiramate	Consider pausing breastfeeding for a couple of days to see if the condition improves.
Two months old infant born underweight. Lack of weight gain.	Lamotrigine	Be observant for rashes, fatigue and poor suction.
Four weeks old infant born prematurely. Drowsy.	Carbamazepine, codeine	Occasional breastfeeding is not dissuaded. Replace some meals with infant formula.
Neonate born five days ago. Drowsy, extrasystoles, feeding problems.	Levetiracetam	Measure the child’s serum concentration of the drug as well as the drug concentration in breast milk.
A few weeks old infant. Shifty eyes, lack of weight gain.	Levetiracetam	Investigate for other causes. Measure the child’s serum concentration of the drug. Consider pausing breastfeeding for a couple of days to see if the condition improves.
Three months old child. Flat growth line.	Levetiracetam	Measure the child’s serum concentration of the drug.
Three weeks old infant. Twitches.	Valproate, lamotrigine	Assess the child’s symptoms on a regular basis.
Five months old child. Twitching during falling asleep.	Lamotrigine	Investigate for other causes. If necessary, introduce solid food considered the child’s age.
Four months old infant. Tremors. Stiffness in leg muscles.	Lamotrigine	Report as suspected adverse reaction. Investigate for other causes.

RELIS = Regional Medicines Information and Pharmacovigilance Centres.

1 In one enquiry the enquirer requested general information about the type of adverse reactions previously observed in infants of mothers who had used levetiracetam during breastfeeding without reference to an individual case.

2 Extracts from answers which represent the main advice from RELIS.

## 4 Discussion

The study unveiled healthcare professionals’ encountered information need related to infants’ safety when breastfed by ASM-using PWE. Most enquirers were physicians, mainly hospital-employed, and nurses, in that order, and they most often called for general information about the compatibility of a given drug therapy with breastfeeding. Decision support with respect to lamotrigine and levetiracetam, alone or in combination, also with other drugs, was most often requested. The enquirer’s uncertainty could directly be caused by restricted or ambiguous drug information advice in official Norwegian drug information sources. A striking finding was that half of the enquiries were put forward after birth, some of them motivated by suspected adverse drug reactions in the infant. The description of healthcare professionals’ challenges concerning breastfeeding in PWE who use ASM can be of value in the development of an adapted information strategy.

### 4.1 Enquirers and enquiries

The main enquirers were physicians and nurses. This is consistent with data from Jahnsen et al. who investigated enquiries related to all types of drug use during breastfeeding [21]. Compared to the percentage of hospital-employed physicians among all physicians posing questions related to all medicine-related questions to RELIS [19], the percentage of hospital employed physicians in our study was substantially higher. Whether physicians outside the hospital had posed their question to a physician in the hospital who then contacted RELIS or whether specialized physicians needed decision support themselves is not evident from our data. Equally, we could not specify the speciality of the enquiring physician by type or numbers. In 2022, nurses accounted for 8 % of all enquiries to RELIS, preceded by physcians and pharmacists [19]. The high percentage of enquiries initiated by nurses in our study may be attributed to the collaborative care model employed in Norway, which includes nurses educated as midwives to provide pregnancy control and postpartum follow-up for both mother and infant [15].

Most enquiries were categorized to be of general nature related to the compatibility of a specific ASM with breastfeeding. The predominance of general enquiries in our material may apply to the experienced overall complexity of managing risk in prescribing drugs for breastfeeding patients [25].

### 4.2 Time point of enquiry

Notably, approximately half of the questions were posed after birth, of which half were asked after initiation of breastfeeding. In a similar study that investigated questions to EURAP in the Netherlands, 28 % of all enquiries were made after birth [26]. Monthly check-ups with a neurologist and regular pregnancy check-ups with a general practitioner/midwife are recommended, according to the Norwegian guidelines for treatment of PWE [15]. Ideally, issues about ASMs and breastfeeding should be addressed prior to birth. Enquiries made after birth or after the initiation of breastfeeding were related to suspected adverse drug reactions in the infant, or to whether drug exposure during breastfeeding was compatible with the infant's state of health. In most cases, however, a reason for the enquiry other than the need for general information was not readily apparent.

Among the enquiries made after birth, 58.9 % lacked information on the child’s state of health. Knowledge of the infant’s health is crucial to assess infant safety in relation to drug use in lactating patients. Impaired health and prematurity can slow down the child’s ability to metabolize and eliminate drugs transferred through breast milk and induce drug-related effects in the infant. The child’s age is also essential because newborns’ capacity for hepatic metabolism and renal elimination is lower compared to infants older than 1 – 2 months [27].

### 4.3 Drugs

Two thirds of the questions involved lamotrigine and levetiracetam, either alone or as part of a polytherapeutic regimen, which is in line with pevious data [26]. These two AMSs are the most frequently used in pregnant and lactating PWE, with lamotrigine even being the ASM of first choice in fertile PWE [28], [29]. The SPCs advise to carefully consider risk versus benefit of breastfeeding, and abstain from breastfeeding for lamotrigine and levetiracetam, respectively [30], [31].

Co-medication was a matter of concern in 18 out of 112 enquiries to RELIS. Among non-ASM drugs, antidepressant were the most often co-administered drugs which is in accordance with depression and anxiety being prevalent comorbidities of epilepsy [32]. Information on the breastfed infants’ safety while exposed to several drugs via breastmilk is mainly based on case reports or case series, and most often no general advice is available. Hence, predicting compatibility of several drugs with breastfeeding is challenging and can cause uncertainty among healthcare preofessionals regarding the safety for the breastfed infant.

### 4.4 Drug information sources

Our investigation showed that physicians were puzzled by restricted, ambiguous, or limited product information advice in the manufacturer-dependent PPC of whether to breastfeed or not during treatment with a specific ASM. The PPC is widely utilized by physicians as a source to drug information [33]. The information in the Norwegian PPC is based on the SPC, which recommendations are generally more restrictive. Due to lack of clinical trials including breastfeeding women, documentation deficits on drug transfer to breastmilk, regulations set by medical authorities and fears of litigations, the drug companies rarely state that medicines are safe to use in pregnancy in the SPCs or other product information [34]. This may cause uncertainty about whether to recommend breastfeeding, leading to the PWE not to initiate or wean off breastfeeding, or to non-adherence to drug treatment. In comparison, in manufacturer-independent resources such as the Drug and Lactation database, LactMed, advice may be less restrictive, and recommendations may include an evaluation of benefit and risk [16], [35]. For instance the SPC does not usually recommend breastfeeding during treatment with levetiracetam [31] whereas a non-regulatory, industry-independent information source recommends breastfeeding, but suggests precautions [36]. Current guidelines and published studies indicate that ASM-treated PWE in general can breastfeed their infant [7], [12], [13], [15]. However, safety data is incomplete and mainly based on small studies, case reports and clinical experience. Hence, counselling is recommended to be individualized based upon the up to date knowledge on infant safety of the ASM in question [37].

### 4.5 Drug safety

We found that 10.7 % of the enquiries involved suspected adverse drug reactions in the infant. Lamotrigine and levetiracetam were the most often suspected ASMs to be associated with the reported symptoms in the infant. Whether the observed symptoms in the breastfed infant were related to an effect of the drug(s) in question or were unrelated to the lactating patient’s drug use is not evident from our data. Also, Information on the child’s state of health was sparse or lacking in 33 (58.9 %) postnatally asked questions.

In general, blood concentrations of ASMs in breastfed infants were low compared with the maternal ASM blood concentrations [14] and serious acute adverse reactions from drugs in breastmilk appear to be uncommon [38]. Moreover, exposure to lamotrigine via breastmilk seems to be well tolerated, as is also the case for levetiracetam [36], [39]. It must also be kept in mind that ASMs in two different studies were among the drugs often suspected to be responsible for drug-induced adverse reactions [38], [40]. In our study material, most enquiries regarding suspected adverse drug reactions were related to infants under 2 months of age, which is consistent with previously published findings [38].

### 4.6 Strengths and limitations

To the best of our knowledge, this study is the first to analyze enquiries posed to a Norwegian drug information service regarding the use of ASMs in breastfeeding PWE. The material includes all enquiries about breastfeeding and the use of ASMs from 1995 to 2021 in the RELIS database, and thus reflects the width of encountered drug-related enquiries associated with breastfeeding in ASM-using PWE. Previously answered enquiries are freely available on RELIS’ website. Hence, RELIS itself functions as a source to drug information, probably affecting the number of retrieved enquiries from the database. Furthermore, enquiries to RELIS are spontaneous, and do not necessarily represent drug problems perceived by the general population of healthcare professionals.

New knowledge regarding ASMs and breastfeeding in the last 20–25 years has contributed to changes in recommendations and impacted trends in the use of ASMs among breastfeeding PWE. Hence, more recent enquiries are considered most relevant in designing an information strategy for healthcare professionals who are involved in the follow up and treatment of breastfeeding PWE.

The size of the study group (N = 112) is small. This limits the ability to draw conclusions when testing associations between variables. Additionally, the material in this study is naturalistic and descriptive with risk of biased interpretation. There was no opportunity to clarify any ambiguities in the enquiry, as the study is retrospective in nature and based on already collected material. However, the data material was reviewed multiple times and categorized by one author, with doubtful cases reviewed by all authors, ensuring quality in the study.

## Conclusions

Future drug information strategies should be directed towards the groups of healthcare professionals involved in the follow up of pregnant and lactating PWE. They may include developing a pamphlet directed at physicians and nurses caring for women that gives cohesive answers to the most common questions. Information should also include advice on appropriate online sources of information regarding the use of drugs during breastfeeding, as well as a reminder of the importance to plan drug treatment during the breastfeeding period before the child is born.

## Funding

This research did not receive any specific grant from funding agencies in the public, commercial, or not-for-profit sectors.

## Statement of authors’ contributions to manuscript

BR, KH, and JS had the idea for the study and developed the design, BR, KH, and SRR validated the data and were responsible for data analysis. BR, KH, SRR and JS interpreted the results. BR and KH supervised the work, SRR wrote the first draft of the paper, BR, KH, SRR and JS critically revised the manuscript. BR was responsible for the final content. All authors have read and approved the final manuscript.

## Declaration of Competing Interest

The authors declare that they have no known competing financial interests or personal relationships that could have appeared to influence the work reported in this paper.
