# Supplementary material for: Healthcare professionals’ information need related to antiseizure medication use in breastfeeding patients with epilepsy. Retrospective analysis of enquiries to Norwegian medicines information and pharmacovigilance centers
Source: Epilepsy Behav Rep. 2023 Oct 25;24:100629. doi: 10.1016/j.ebr.2023.100629 (PMC10632412; doi:10.1016/j.ebr.2023.100629)
Supplement: Supplementary data 1 [file mmc1.docx]

**Table A.1 – Variables, categories, and rules of interpretation**

| **Variabel** | **Categories** | **Rules of interpretation, comments** |
| --- | --- | --- |
| RELIS center | 1 = South-East  2 = West  3 = Mid  4 = North |  |
| Questioner’s health care profession | 1 = Physician  2 = Nurse  3 = Pharmacist  4 = Other | - 2: Including midwife and public health nurse |
| Physician’s place of employment | 1 = Physician in hospital  2 = Physician outside hospital | - 2: Including regular general practitioner/family doctor and doctor at a health care station |
| Topic of the query | 1 = Requesting general information regarding specific medication(s).  2 = Uncertain because the Norwegian Pharmaceutical Product Compendium or product information advise against use of the actual medication  3 = Requesting updated assessment from RELIS  4 = Adverse effects in breastfed infant  5 = Uncertain due to treatment with ASMs  6 = Uncertain due to treatment with ASM in combination with other mediation  7 = Other |  |
| Treatment with more than one ASM | 0 = Unknown, not registered  1 = Yes  2 = No | - 0: It is not explicitly stated in the question whether the patient uses more than one antiseizure medication (e.g., the enquirer questions only one ASM, but it is not mentioned/denied that the patient is treated with other ASM). - 2: It is explicitly stated in the question that the patient is not treated with more than one ASM. |
| Specify type of antiseizure medication | {1, phenobarbital} etc. See table A.2 for categorization of all ASMs | - Each ASM is registered according to appendix 3. - If several ASMs are assessed in the same QAP, these ASMs are registered separately, but with the same question number and RELIS center. |
| Treatment with other medication(s) in addition to ASM | 0 = Unknown, not registered  1 = Yes  2 = No | - 0: It is not explicitly stated in the question whether the patient uses other medication(s) in addition to ASM (e.g., it is not mentioned/denied that the patient uses other medication). - 2: It is explicitly stated in the question that the patient is not treated with other medication in addition to ASM. |
| Timing of the question relative to breastfeeding initiation | 0 = Unknown  1 = Before initiated breastfeeding  2 = After initiated breastfeeding |  |
| Timing of the question relative to birth | 1 = Before birth  2 = After birth |  |
| Child’s age mentioned in the question | 1 = Yes  2 = No 3 = Not born | - 1: The child’s age appears in the question’s wording. |
| The child’s age if this is mentioned | 1 = 0 – 2 months 2 = > 2 months | - 1: Examples of wording: “recently born”, “born a few days ago” etc. |
| Information about the child’s state of health | 1 = Yes  2 = No  3 = Not born | - 1: The child’s general state of health is described in the question. Inquiries where possible side effects in a breastfed child are reported (e.g., twitching, rashes etc.) are categorized as “yes” even if the general state of health is not mentioned. |
| The child’s state of health if this is mentioned | 1 = Healthy  2 = Disability/illness/premature | - 2: Including inquires where possible side effects in a breastfed child are reported (e.g., twitching, rashes etc.). |
| RELIS’ conclusion regarding compatibility with breastfeeding | 0 = No specific advice given  1 = Compatible with breastfeeding. Even if there is information about possible side effects in breastfed infants when it appears that breastfeeding is still recommended.  2 = Take precautions. If so, compatible with breastfeeding  3 = Breastfeeding is not recommended | - 2: Examples of precautions: Take medication immediately after breastfeeding, monitoring the child in respect of specific symptoms, measuring the child’s serum concentration. - If the answer only refers to another RELIS-inquiry, the conclusion in this inquiry is used for categorization. |

ASM = antiseizure medication, QAPs = question-and-answer pairs. RELIS = regional medicines and pharmacovigilance centers

**Table A.2 – Overview and categorization of antiseizure medications**

| **Category** | **ATC-number** | **Generic name** |
| --- | --- | --- |
| 1 | N03A A02 | Phenobarbital |
| 2 | N03A B02 | Phenytoin |
| 3 | N03A D01 | Ethosuximide |
| 4 | N03A E01 | Clonazepam |
| 5 | N03A F01 | Carbamazepine |
| 6 | N03A F02 | Oxcarbazepine |
| 8 | N03A F03 | Rufinamide |
| 7 | N03A F04 | Eslicarbazepin |
| 9 | N03A G01 | Valproate |
| 10 | N03A G04 | Vigabatrin |
| 11 | N03A X09 | Lamotrigine |
| 12 | N03A X10 | Felbamate |
| 13 | N03A X11 | Topiramate |
| 14 | N03A X12 | Gabapentin |
| 15 | N03A X14 | Levetiracetam |
| 16 | N03A X15 | Zonisamide |
| 17 | N03A X16 | Pregabalin |
| 18 | N03A X 17 | Stiripentol |
| 19 | N03A X18 | Lacosamide |
| 20 | N03A X22 | Perampanel |
| 21 | N03A X23 | Brivaracetam |
| 22 | N03A X24 | Cannabidiol |
| 23 | N05B A09 | Clobazam |
| 24 | N03A G06 | Tiagabine |
| 25 | N03A X03 | Sulthiame |
